# Low neutrophil alkaline phosphatase score is a new aspect of calreticulin-mutated myeloproliferative neoplasms

**DOI:** 10.1186/s40064-016-2829-6

**Published:** 2016-07-22

**Authors:** Toshinori Kondo, Taizo Tasaka, Nanako Tomioka, Fuminori Sano, Hirotoshi Tokunaga, Shin-ichiro Suemori, Takayuki Tsujioka, Yoshiko Matsuhashi, Hidekazu Nakanishi, Hideho Wada, Kaoru Tohyama, Takashi Sugihara

**Affiliations:** Department of Hematology, Kawasaki Medical School, 577 Matsushima, Kurashiki City, Okayama Prefecture 701-0192 Japan; Department of Laboratory Medicine, Kawasaki Medical School, 577 Matsushima, Kurashiki City, Okayama Prefecture 701-0192 Japan; Department of Transfusion Medicine and Cell Therapy, Saitama Medical Center, Saitama Medical University, 1981 Kamoda, Kawagoe City, Saitama Prefecture 350-8550 Japan

**Keywords:** Myeloproliferative neoplasms, Calreticulin, Alkaline phosphatase score, Essential thrombocythemia, Janus kinase 2

## Abstract

Calreticulin (*CALR*) and *JAK2*-V617F gene mutations, which are major genetic mutations in patients with primary myelofibrosis (PMF) and essential thrombocythemia (ET), exert different effects on the clinical features and outcomes of these diseases. We analyzed 88 and 9 patients with ET and PMF, respectively, and determined the differences in the clinical characteristics of ET patients with *JAK2*-V617F compared with *CALR* mutations. The frequency of the *JAK2*-V617F and *CALR* mutations were 64 and 22 %, respectively. Patients with *CALR* mutations were younger, had a lower white blood cell count, and had a lower rate of thrombotic events than patients with the *JAK2* mutation. The neutrophil alkaline phosphatase (NAP) score of 16 patients with *CALR* mutations was significantly lower than the normal controls, which was mainly due to the high proportion of NAP-negative neutrophils. This is the first report to show an association between *CALR* mutations in patients with myeloproliferative neoplasms (MPN) and the NAP score. Although the mechanism is unclear, the NAP score could be a useful and reliable biochemical marker to discriminate the mutational status of MPN patients. Further investigation is warranted to determine whether these characteristics contribute to the pathogenesis of MPN and the NAP score.

## Background

The pathogenesis of myeloproliferative neoplasms (MPN) is characterized by constitutive activation of “disease-specific” protein tyrosine kinases. The Janus kinase 2 (*JAK2*)-V617F mutation and somatic mutations of *JAK2* exon 12 constitutively activate JAK2 protein tyrosine kinase and these mutations are detected in almost all patients with polycythemia vera (PV). In addition, the *JAK2*-V617F mutation is detected in approximately 50–60 % of patients with essential thrombocythemia (ET) and primary myelofibrosis (PMF) (Baxter et al. 2005; James et al. 2005; Kralovics et al. 2005; Levine et al. 2005; Campbell and Green 2006), and activating mutations of the myeloproliferative leukemia virus oncogene (*MPL*) are present in 5–10 % of patients with ET or PMF without the *JAK2*-V617F mutation (Pikman et al. 2006; Rumi et al. 2013). Furthermore, novel mutations in the gene encoding calreticulin (*CALR*) were discovered in patients with ET and PMF with wild-type *JAK2* or *MPL* (Klampfl et al. 2013; Nangalia et al. 2013). *CALR* mutations are detected only in patients with ET or PMF (Klampfl et al. 2013; Nangalia et al. 2013). The frequency of *CALR* mutations in patients with ET and PMF are 25 and 35 %, respectively. The clinical features of ET patients with *CALR* mutations include a higher platelet count, lower hemoglobin level and white blood cell count, fewer thrombotic events, and less leukemic transformation compared to ET patients with *JAK2*-V617F mutation (Rumi et al. 2014). These results suggest that the clinical features and outcomes of MPN patients are defined by the gene alterations that they acquired.

The neutrophil alkaline phosphatase (NAP) score is a useful and reliable biochemical marker that supports the diagnosis of MPN (Bendix-Hansen and Bergmann 1985). The chronic phase of chronic myelogenous leukemia (CP-CML) is characterized by a low NAP score that is elevated when the disease enters the accelerated phase (Kaplow 1971). Some MPN patients with *JAK2*-V617F mutation have higher NAP scores. Although the NAP score often reflects the pathophysiological status of patients with MPNs, to our knowledge, the details of NAP scores of patients with MPN with *CALR* mutations have never been studied. Therefore, the goal of the present study was to identify the clinical features of MPN patients with *JAK2*-V617F and *CALR* mutations by focusing on their NAP scores.

## Methods

### Patients

We recruited 88 patients with ET and 9 with PMF. Patients were diagnosed according to the criteria of the 2008 WHO classification. Clinical laboratory findings acquired at the first visit were utilized. The ethics committees of Kawasaki Medical School and Kawasaki Medical School Hospital (Kurashiki, IRB No. 1747 and 1769) approved this study, and all patients provided written informed consent.

### NAP score analysis

The NAP score was determined using a peripheral blood smear stained using our laboratory’s protocol. Briefly, slides with dried peripheral blood smears were fixed for 5 s in ice-cold methanol containing 10 % formalin and 0.001 % glacial acetic acid. After drying, the slides were treated with a staining solution (0.13 mmol/l naphthol AS-MX phosphate, 0.25 mol/l dimethylformamide, 0.076 mol/l propanediol buffer, and 0.04 mmol/l Fast Blue RR Salt) at 37 °C for 2 h. After washing with water, the slides were stained using 1 % safranin for 2 min. Neutrophils (*n* = 100) were assigned scores ranging from 0 to 5 according to the number and distribution of NAP-positive granules. The median NAP score of a healthy control in our laboratory is 250.

### DNA sequence analysis

Genomic DNA was isolated from polymorphonuclear leukocytes of patients’ peripheral blood. We tested for *JAK2*, *MPL*, and *CALR* mutations that were previously reported in patients with ET or PMF (Kondo et al. 2008). The primers, which were designed using Primer3 (version 0.4.0) software (accessible at http://primer3.sourceforge.net/), used to detect the mutations were as follows (forward, reverse): *JAK2*-V617F, 5′-AGTCATGCTGAAAGTAGGA-3′ and 5′-ATTGCTTTCCTTTTTCACAAGAT-3′; *CALR* exon 9, 5′-CTGGTCCTGGTCCTGATGTC-3′ and 5′-CAGAGACATTATTTGGCGCG-3′; and *MPL* exon 10, 5′-AGAGTAGGGGCTGGCTGGAT-3′ and 5′-CAGGTCCCACCTCCTAAACC-3′. DNA was amplified using polymerase chain reaction (PCR) Master Mix (Promega KK, Madison, WI, USA) as follows: 1 cycle at 95 °C for 2 min, 35 cycles of denaturation at 95 °C for 30 s, annealing at 52 °C (*MPL*, 60 °C) for 30 s, and extension at 72 °C for 30 s. PCR products were analyzed using 3 % agarose gel electrophoresis in the presence of ethidium bromide, and the amplicons were purified using Illustra ExoProStar (GE Healthcare Life Sciences, Buckinghamshire, UK). Direct sequencing was performed using the Big Dye Terminator ver3.1 Cycle Sequencing Kit (Applied Biosystems, Foster City, CA, USA) and an ABI Prism 3130 Genetic Analyzer. Sequence data were analyzed using Mutation Surveyor ver3.10 (Softgenetics, State College, PA, USA).

Subjects’ characteristics and clinical parameters were compared using the *χ*^2^ test, Mann–Whitney *U* test, and Kruskal–Wallis tests. The strength of the association between two variables was determined using the Spearman’s rank correlation. Probability values <0.05 were considered significant. Statistical analyses were performed using SPSS ver.15.0.1 J software (IBM, Tokyo, Japan).

## Results

We searched for *JAK2*, *CALR*, and *MPL* mutations in 88 patients with ET and 9 patients with PMF. Table 1 shows the clinical and laboratory features at diagnosis of the patients stratified according to MPN subtype and mutational status. In the 88 patients with ET, the frequency of the *JAK2*-V617F and *CALR* exon-9 mutations were 65 and 21 %, respectively, and in the patients with PMF, they were 56 and 22 %, respectively. *MPL* mutations were not detected in any patient. Triple negativity for *JAK2*, *CALR*, and *MPL* mutations was found in 14 and 22 % of the patients with ET and PMF, respectively. *CALR* mutations were detected in 21 patients, and the details of these mutations are shown in Table 2. All mutations were heterogeneous insertions or deletions in exon 9, with 10 distinct variants as follows: six deletions, two insertions, and two complex insertions and deletions. All *CALR* mutations were predicted to generate a commonly known C-terminal peptide sequence (Klampfl et al. 2013; Nangalia et al. 2013). There were two common variants: L367 fs*46 (33 %) and K385 fs*47 (24 %).

**Table 1:** Presenting features of the 97 patients with ET or PMF, stratified according to their mutational status

	*JAK2* mutated (A)	*CALR* mutated (B)	Triple negative	*p* (A vs B)*
ET (N = 88)				
No. of patients, n (%)	57 (65)	19 (21)	12 (14)	
Age, median (range), year	66 (33–86)	58 (30–85)	66.5 (29–82)	0.056
Age ≧ 60 year, n (%)	42 (74)	8 (42)	8 (67)	0.012
Females, n (%)	33 (58)	9 (47)	5 (42)	0.439
Leukocytes, median (range), ×10^9^/L	12.1 (6.0–42.3)	9.2 (5.3–16.3)	8.4 (4.6–13.9)	<.001
Hemoglobin, median (range), g/dL	14.3 (10.2–18.6)	13.5 (11.4–17.1)	13.7 (11.3–16.2)	0.593
Platelets, median (range), ×10^9^/L	867 (489–2285)	930 (631–2336)	717 (501–922)	0.359
NAP score median (range)	312 (202–417)	215 (106–286)	254.5 (116–404)	<.001
High LD, n (%)	38 (67)	11 (58)	3 (25)	0.489
Palpable splenomegaly, n (%)	19 (33)	3 (16)	1 (8)	0.144
Thrombosis history, n (%)	22 (39)	2 (11)	3 (25)	0.023
Needed to cytoreductive therapy, n (%)	39 (68)	12 (63)	5 (42)	0.672
PMF (N = 9)				
No. of patients, n (%)	5 (56)	2 (22)	2 (22)	
Age, median (range), year	65 (51–85)	63.5 (58–69)	67.5 (57–78)	NA
Age ≧ 60 year, n (%)	4 (80)	1 (50)	1 (50)	NA
Females, n (%)	3 (60)	1 (50)	1 (50)	NA
Leukocytes, median (range), ×10^9^/L	14.4 (8.6–37.1)	5.8 (5.6–6.1)	5.1 (5.0–5.3)	NA
Hemoglobin, median (range), g/dL	13.2 (9.8–16.8)	12.2 (11.7–12.6)	11.1 (8.9–13.3)	NA
Platelets, median (range), ×10^9^/L	384 (194–654)	306 (260–352)	383 (122–644)	NA
NAP score median (range)	341 (301–378)	157 (150–164)	253.5 (210–297)	NA
High LD, n (%)	5 (100)	2 (100)	1 (50)	NA
Palpable splenomegaly, n (%)	5 (100)	2 (100)	0 (0)	NA
Thrombosis history, n (%)	1 (20)	0 (0)	1 (50)	NA
Needed to cytoreductive therapy, n (%)	1 (20)	0 (0)	1 (50)	NA

NA Not available

*Calculated using the χ^2^ test except for age, CBC and NAP score, which was calculated using the Mann–Whitney *U* test

**Table 2:** Mutational status of CALR exon 9 in the 21 patients with ET or PMF

Diagnosis	cDNA change	Protein change	C-terminal novel amino acid sequence
ET	c.1099_1150del	p.L367 fs*46	TRRMMRTKMRMRRMRRTRRKMRRKMSPARPRTSCREACLQGWTEA
ET	c.1099_1150del	p.L367 fs*46	TRRMMRTKMRMRRMRRTRRKMRRKMSPARPRTSCREACLQGWTEA
PMF	c.1099_1150del	p.L367 fs*46	TRRMMRTKMRMRRMRRTRRKMRRKMSPARPRTSCREACLQGWTEA
ET	c.1092_1143del	p.L367 fs*46	TRRMMRTKMRMRRMRRTRRKMRRKMSPARPRTSCREACLQGWTEA
ET	c.1099_1150del	p.L367 fs*46	TRRMMRTKMRMRRMRRTRRKMRRKMSPARPRTSCREACLQGWTEA
ET	c.1099_1150del	p.L367 fs*46	TRRMMRTKMRMRRMRRTRRKMRRKMSPARPRTSCREACLQGWTEA
ET	c.1099_1150del	p.L367 fs*46	TRRMMRTKMRMRRMRRTRRKMRRKMSPARPRTSCREACLQGWTEA
ET	c.1154_1155ins TTGTC	p.K385 fs*47	NCRRMMRTKMRMRRMRRTRRKMRRKMSPARPRTSCREACLQGWTEA
PMF	c.1154_1155ins TTGTC	p.K385 fs*47	NCRRMMRTKMRMRRMRRTRRKMRRKMSPARPRTSCREACLQGWTEA
ET	c.1154_1155ins TTGTC	p.K385 fs*47	NCRRMMRTKMRMRRMRRTRRKMRRKMSPARPRTSCREACLQGWTEA
ET	c.1154_1155ins TTGTC	p.K385 fs*47	NCRRMMRTKMRMRRMRRTRRKMRRKMSPARPRTSCREACLQGWTEA
ET	c.1154_1155ins TTGTC	p.K385 fs*47	NCRRMMRTKMRMRRMRRTRRKMRRKMSPARPRTSCREACLQGWTEA
ET	c.1102_1153del	p.K368 fs*45	RRMMRTKMRMRRMRRTRRKMRRKMSPARPRTSCREACLQGWTEA
ET	c.1102_1153del	p.K368 fs*45	RRMMRTKMRMRRMRRTRRKMRRKMSPARPRTSCREACLQGWTEA
ET	c.1110_1140del	p.E371 fs*49	RQRTRRMMRTKMRMRRMRRTRRKMRRKMSPARPRTSCREACLQGWTEA
ET	c.1109_1160del	p.E370 fs*42	VMRTKMRMRRMRRTRRKMRRKMSPARPRTSCREACLQGWTEA
ET	c.1150_1154delins TGTC	p.D384 fs*46	CRRMMRTKMRMRRMRRTRRKMRRKMSPARPRTSCREACLQGWTEA
ET	c.1125_1126 ins TTCTTAGTGCT	p.R376 fs*58	FLVLAKRRRRQRTRRMMRTKMRMRRMRRTRRKMRRKMSPARPRTSCREACLQGWTEA
ET	c.1091_1124del	p.E364 fs*54	DAKRRRRQRTRRMMRTKMRMRRMRRTRRKMRRKMSPARPRTSCREACLQGWTEA
ET	c.1080_1144delins GGAAGAAGACAAG	p.Q361 fs*51	KAAEKQMKDKEEDKQRTRRMMRTKMRMRRMRRTRRKMRRKMSPARPRTSCREACLQGWTEA
ET	c.1122_1134del	p.K375 fs*50	KAAEKQMKDKQDEEQRLKEEEEDKRRRQRTRRMMRTKMRMRRMRRTRRKMRRKMSPARPRTSCREACLQGWTEA

We investigated the clinical features of ET patients with *JAK2* and *CALR* mutations. The median age of ET patients with *CALR* mutations was less than the ET patients with *JAK2* mutations, although this difference was not significant (*p* = 0.056). The proportion of patients aged >60 years was significantly lower in the *CALR* mutation group (*p* = 0.012). The ET patients with *CALR* mutations had a significantly lower white blood cell count (*p* < 0.001), NAP score (*p* < 0.001), and prevalence of thrombotic events than the patients with *JAK2* mutations (*p* = 0.023; Table 1).

**Fig. 1:**
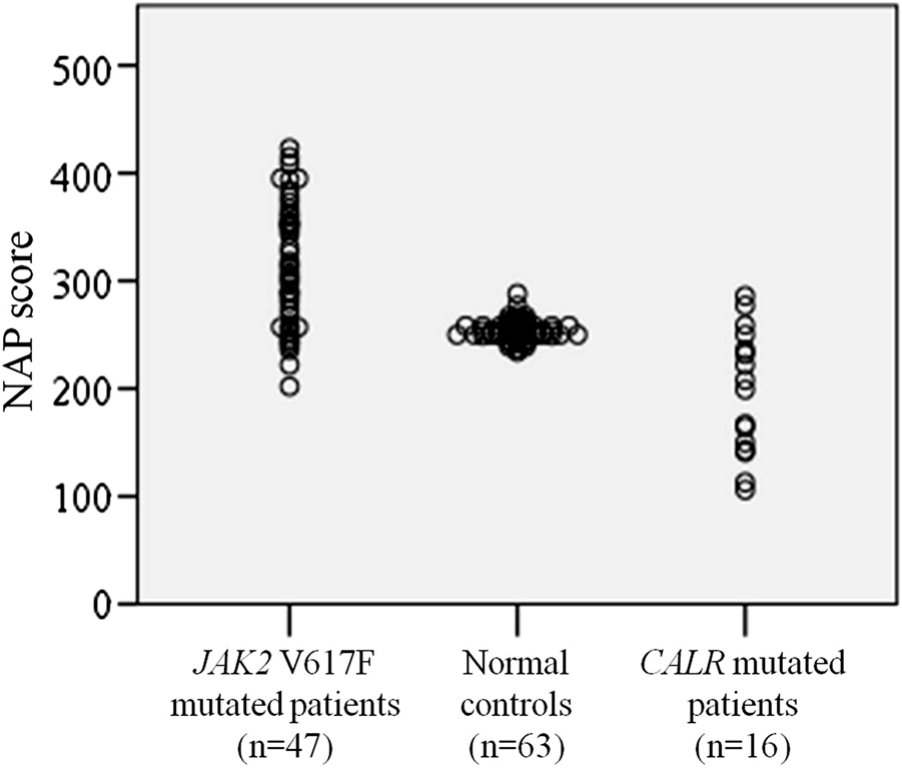
Neutrophil alkaline phosphatase scores of patients with essential thrombocythemia or primary myelofibrosis stratified according to their mutational status. The probability was calculated using the Kruskal–Wallis test (*p* < 0.001)

**Fig. 2:**
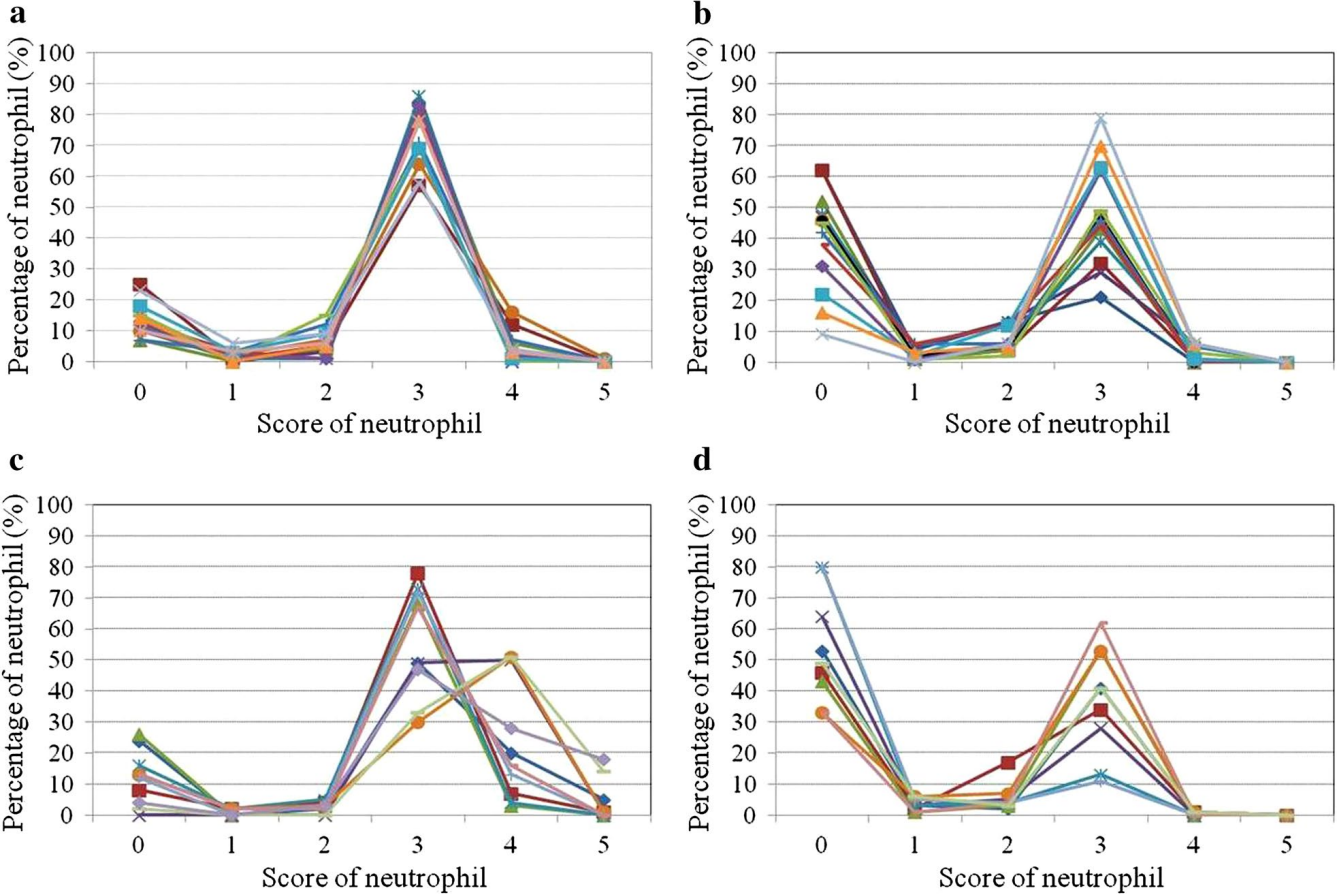
Analysis of the neutrophil alkaline phosphatase rate according to the mutational status of patients with MPN. **a** Normal controls (*n* = 14). **b** Patients with MPN with *CALR* mutations (*n* = 13). **c** Patients with MPN with *JAK2*-V617F mutation (*n* = 10). **d** Patients in the CP-CML (*n* = 9). One hundred neutrophils were counted in each sample

The NAP scores of 63 patients with MPN were evaluable, including 47 patients with *JAK2* mutations and 16 patients with *CALR* mutations. The median NAP score of the patients with *CALR* mutations was significantly lower than normal controls (Fig. 1). In contrast to the normal to high range of NAP scores of the patients with *JAK2* mutations, the scores of the patients with *CALR* mutations were normal to low. The NAP rate of the MPN patients with *CALR* mutations was lower (*n* = 13; median NAP rate 55 %; range 38–91 %), mainly due to the high proportion of NAP-negative neutrophils (score = 0; Fig. 2). This pattern of NAP scores was similar to that of patients with CP-CML with low NAP scores (*n* = 9; median NAP rate 51 %; range 20–67 %; Fig. 2). There was no significant correlation between the NAP score and any clinical parameter (white blood cell count, hemoglobin value, platelet count, or disease duration) in the MPN patients with *CALR* mutations (data not shown). The NAP rate of the MPN patients with *JAK2* mutations was high compared with patients with *CALR* mutations (*n* = 10; median 87.5 %; range 74–100 %) and may be explained by the increase in strongly NAP-positive neutrophils (score 4 or 5). In patients with *JAK2* mutations, the NAP score was significantly correlated with the burden of the V617F mutant allele (*n* = 47, *p* < 0.001, *r* = 0.748).

## Discussion

In 2013, two groups reported the discovery of *CALR* mutations in patients with ET or PMF (Klampfl et al. 2013; Nangalia et al. 2013). In the present study, the frequencies of *CALR* mutations in patients with ET and PMF were 21 and 22 %, respectively. The frequencies of *CALR* L367 fs*46 and K385 fs*47 mutations, which are the most common mutations in Western countries (Klampfl et al. 2013; Nangalia et al. 2013), were 33 and 24 %, respectively, in our series. Patients with *CALR* mutations are younger, have a lower hemoglobin level and white blood cell count, and have a higher platelet count and serum erythropoietin than patients with *JAK2*-V617F mutation (Rumi et al. 2014). *CALR* mutations can also affect the clinical outcomes. ET patients with *CALR* mutations do not transform to PV (Rumi et al. 2014). In our series, the clinical features of the patients with *CALR* mutations included younger age, decreased white blood cell count, and a lower rate of thrombosis compared with patients with *JAK2* mutations, although there were no significant differences in hemoglobin levels or platelet count. Further studies are required to better understand the phenotypes of patients with *CALR* mutations. Altogether, the available data suggest that the differences in mutational status define the clinical subtypes of the disease.

We found that the NAP score of patients with *JAK2* mutations generally ranged from normal to high levels and was correlated with their *JAK2*-V617F mutant-allele burden. In contrast, the median NAP score of the 16 patients with *CALR* mutations was significantly lower than the normal controls. To our knowledge, this is the first study to report low NAP scores in patients with *CALR* mutations, which were mainly due to the high proportion of NAP-negative neutrophils and represents a new biological aspect of MPN patients with *CALR* mutations. Therefore, the NAP score could be a useful and reliable biochemical marker to discriminate the mutational status in patients with ET or PMF.

In patients with CP-CML, a low NAP score is the result of low serum levels of granulocyte colony-stimulating factor (Yuo et al. 1987; Watari et al. 1989; Saitoh and Shibata 1993; Tsushita et al. 1993). In contrast, in the present study, there was no significant correlation between these variables (data not shown). Several reports describe the relationship between NAP activation and MPN-related mutations. For example, transfection of the NB4 cell line with a construct that expresses *JAK2*-V617F induces cell proliferation through the phosphorylation of the signal transducer and activator of transcription (STAT) 5, leading to phosphorylation of STAT3, which activates NAP expression (Oku et al. 2010). Furthermore, patients with MPN, regardless of their diagnosis or *JAK2* mutational status, are characterized by upregulation of *JAK*-*STAT* signaling molecules, and the *CALR* mutation activates the *JAK2*-*STAT5* signaling pathway (Rampal et al. 2014). A recent report suggested that *JAK2* signaling might occur in ET megakaryocytes with *CALR* mutations; however, the downstream signaling mechanisms other than those involving STAT3 and STAT5 are likely involved in the pathogenesis of MPN with *CALR* mutations (Lau et al. 2015). Finally, STAT1, STAT3, and STAT5 were not phosphorylated in the MARIMO cell line derived from a patient with ET harboring a *CALR* mutation (Kollmann et al. 2015). Taken together, these findings suggest the possibility that a lack of STAT3 phosphorylation may be related to the lower NAP score observed in MPN patients with *CALR* mutations. In patients with *JAK2*-V617F, NAP expression increases in parallel to the V617F allele burden (Basquiera et al. 2007; Vannucchi et al. 2007); however, the relationship between the *CALR*-mutated allele burden and NAP expression is unknown.

The detailed mechanism that causes *CALR* mutations and their relationship with a low NAP score remains to be elucidated. Our results reinforce the biological and diagnostic importance of the relationship between the NAP score and MPN.

## Conclusion

*CALR* mutations are one of the major driver mutations in ET and PMF. In addition, the NAP score reflects the pathophysiological status of patients with MPN. We found that the NAP score of patients with *CALR* mutations ranged from normal to low levels, and that the median NAP score of patients with *CALR* mutations was significantly lower than the normal controls. This is the first report of the relationship between the NAP score and *CALR* mutations, but the detailed mechanism is still unclear. A low NAP score is a unique aspect of MPN patients with *CALR* mutations.
